# The soil microbiome of *Lolium perenne* L. depends on host genotype, is modified by nitrogen level and varies across season

**DOI:** 10.1038/s41598-024-56353-2

**Published:** 2024-03-08

**Authors:** Cristiana Paina, Mattia Fois, Torben Asp, Just Jensen, Pernille Bjarup Hansen, Palle Duun Rohde

**Affiliations:** 1 Department of Agroecology, Aarhus University, Forsøgsvej 1, 4200 Slagelse, Denmark; 2 Center for Quantitative Genetics and Genomics, Aarhus University, Forsøgsvej 1, 4200 Slagelse, Denmark; 3 Center for Quantitative Genetics and Genomics, Aarhus University, C. F. Møllers Allé 3, Bldg. 1130, 8000 Aarhus, Denmark; 4 Genomic Medicine, Department of Health Science and Technology, Aalborg University, Selma Lagerløfs Vej 249, 9260 Gistrup, Denmark

**Keywords:** Biotechnology, Ecology, Plant sciences

## Abstract

Genotype by environment interactions (G × E) are frequently observed in herbage production. Understanding the underlying biological mechanisms is important for achieving stable and predictive outputs across production environments. The microbiome is gaining increasing attention as a significant contributing factor to G × E. Here, we focused on the soil microbiome of perennial ryegrass (*Lolium perenne* L.) grown under field conditions and investigated the soil microbiome variation across different ryegrass varieties to assess whether environmental factors, such as seasonality and nitrogen levels, affect the microbial community. We identified bacteria, archaea, and fungi operational taxonomic units (OTUs) and showed that seasonality and ryegrass variety were the two factors explaining the largest fraction of the soil microbiome diversity. The strong and significant variety-by-treatment-by-seasonal cut interaction for ryegrass dry matter was associated with the number of unique OTUs within each sample. We identified seven OTUs associated with ryegrass dry matter variation. An OTU belonging to the *Solirubrobacterales* (*Thermoleophilales*) order was associated with increased plant biomass, supporting the possibility of developing engineered microbiomes for increased plant yield. Our results indicate the importance of incorporating different layers of biological data, such as genomic and soil microbiome data to improve the prediction accuracy of plant phenotypes grown across heterogeneous environments.

## Introduction

Perennial ryegrass (*Lolium perenne* L.) is a forage species widely cultivated in temperate grasslands^1^. Grasslands make a significant contribution to human food security by providing fodder for ruminants used for meat and milk production. However, frequent occurrence of genotype by environment interactions (G × E, reflected in the varying response of different genotypes to changing environmental factors) in herbage yield production results in heterogeneous herbage production across years and seasons, which complicates the prediction of yield and quality^2–6^. Understanding the contributing mechanisms that drive inconsistent output for perennial ryegrass yield is essential for optimizing the breeding programs to achieve stable and predictive output across different and variable environments.

The plant microbiome, in general, has gained increasing attention through the past years. It has become evident that the soil microbiome interplays with the plants, affecting both yield and plant performance in a specific environment^7–10^. Often, the host plant relies on the soil microbiome to provide essential nutrients, whereas the plant cultivates its microbiome, for example by releasing specific metabolites in the soil, adjusting the soil pH, and reducing competition among beneficial microbes^7^. Because of this close relationship between plants and the surrounding soil microbiome, it is not surprising that the composition of microbial community varies among plant species^8^. It has been shown that the genotype of the plant is involved in structuring the microbiome of maize^10–12^, *Arabidopsis thaliana*^13–15^, barley^16^, rice^17–19^, wheat^20^. Likewise, certain components of the microbiome have been shown to contribute with enhanced plant resistance towards biotic and abiotic stressors, for example wilt resistance in tomato^21^, or drought resistance in *Capsicum annuum*^22^. The soil microbiome was shown to interfere with physiological processes in the plant, for example altering flowering time through influencing the metabolic network of phytohormones^23^. Moreover, the soil microbiome was suggested to mediate positive relationships between plant diversity and plant productivity^24^. The soil microbiome functions not only as a plant health indicator, but also as an indicator of soil health^25^.

Despite years of research attempting to uncover the association between host plant and the soil microbiome composition^26–28^, little is known about the associated soil microbial community of perennial ryegrass. Chen et al. investigated in a controlled experiment the root-associated bacterial microbes of ryegrass across different compartments of the roots and soil and found differences in bacterial composition across the compartments, as well as across soil types^29^. Understanding the interactions between the host plant and the soil microbiome and eventually identifying the genetic basis controlling such interactions, will be of great significance for plant breeding^30^. The aim of this study was to determine to what extent the soil microbiome varied among 20 ryegrass varieties grown under field conditions, whether the soil microbial community was modified by nitrogen level and how the season affected the soil microbiome community.

## Results

### Composition of the soil microbial community

Across the 240 samples (20 varieties, 2 nitrogen treatments, 3 samplings at seasonal cuts and 2 replicates) a total of 10,010 bacterial OTUs were initially detected, with the number of sequences per sample ranging between 29,710—54,850 (mean 41,850). We detected 6,130 fungal OTUs, with the number of sequences per sample ranging between 37,959—287,558 (mean 131,026). A total of 6,092 bacterial and 2,907 fungal OTUs remained after removing OTUs with frequency < 0.1% (Supplementary Tables S4, S5).

**Figure 1:**
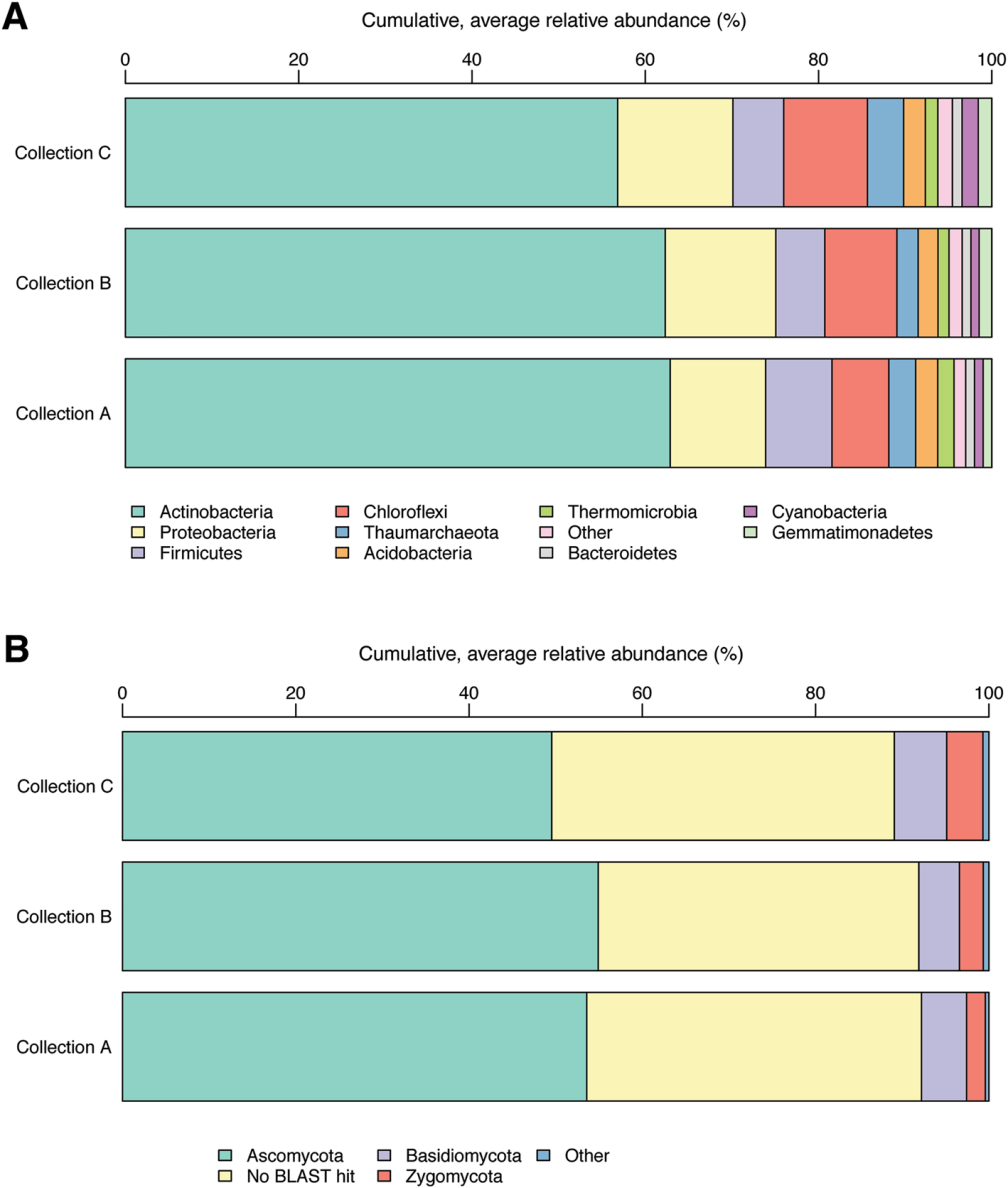
Representation of the (**A**) bacteria and (**B**) fungi composition at phylum level. Each bar represents the relative abundance (%) averaged across samples within collection points (A: 24.05.2017, B: 07.07.2017, C: 17.08.2017). Only taxonomic groups with relative abundance > 1% are shown (remaining is agglomerated in the category “Others”).

The most abundant bacteria phyla were *Actinobacteria*, *Chloroflexi*, *Proteobacteria,* and *Firmicutes* (Fig. 1A), and the most abundant fungi phylum was *Ascomycota*, followed by *Basidiomycota* and *Zygomycota* (Fig. 1B). A considerable proportion of the fungal OTUs had no BLAST hits (Fig. 1B).

**Figure 2:**
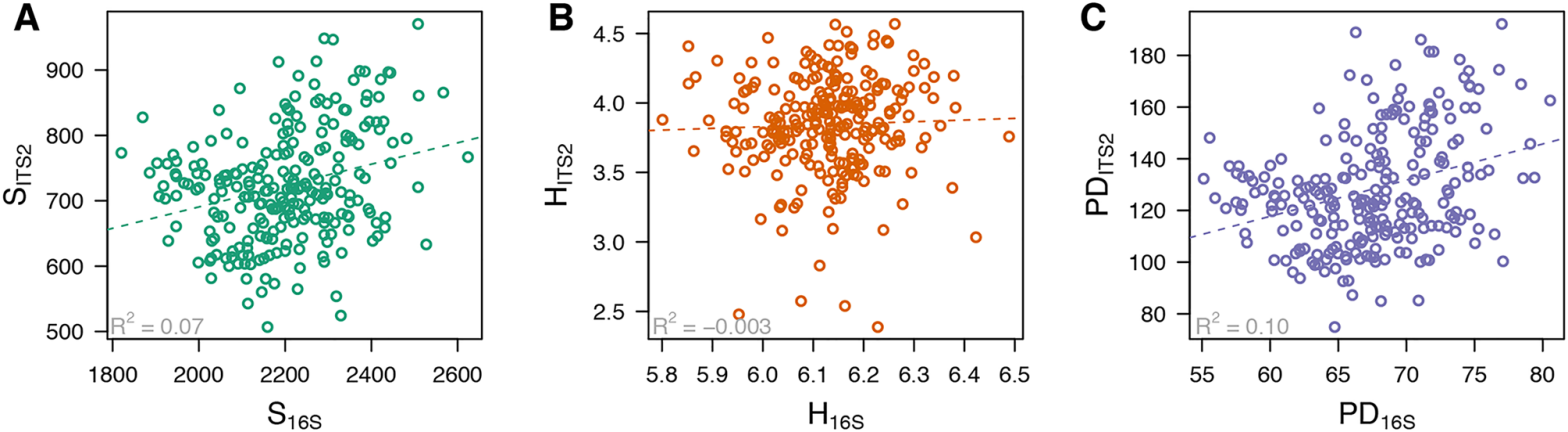
Comparison of α-diversity metrics for the bacterial (16S) and fungal (ITS2) soil microbiome. The three panels show the comparison of (**A**) OTU richness *S*; (**B**) Shannon's index *H*; and (**C**) Phylogenetic diversity *PD*. Variance explained (*R*^2^) by a linear fit is noted.

The OTU richness is the number of unique OTUs within each sample, whereas Shannon’s Index also accounts for both OTU abundance and evenness. Phylogenetic diversity is a measure of biodiversity that incorporates the differences in phylogenetic diversity between OTUs, such that related individuals increase the measure of phylogenetic biodiversity less than unrelated individuals do. The OTU richness and Shannon’s Index were lower for fungi than for bacteria (Fig. 2), indicating that the within-sample diversity of bacteria was larger compared to the fungal community (Supplementary Table S6). Contrary, the phylogenetic diversity was two-fold larger for the fungal community than for bacteria (Fig. 2). These trends are generalized across all samples and comparing the α-diversity measures between bacteria and fungi showed no correlation within samples (Fig. 2), but strong correlation was observed among diversity metrics within bacteria and within fungi (Supplementary Fig. S1).

**Figure 3:**
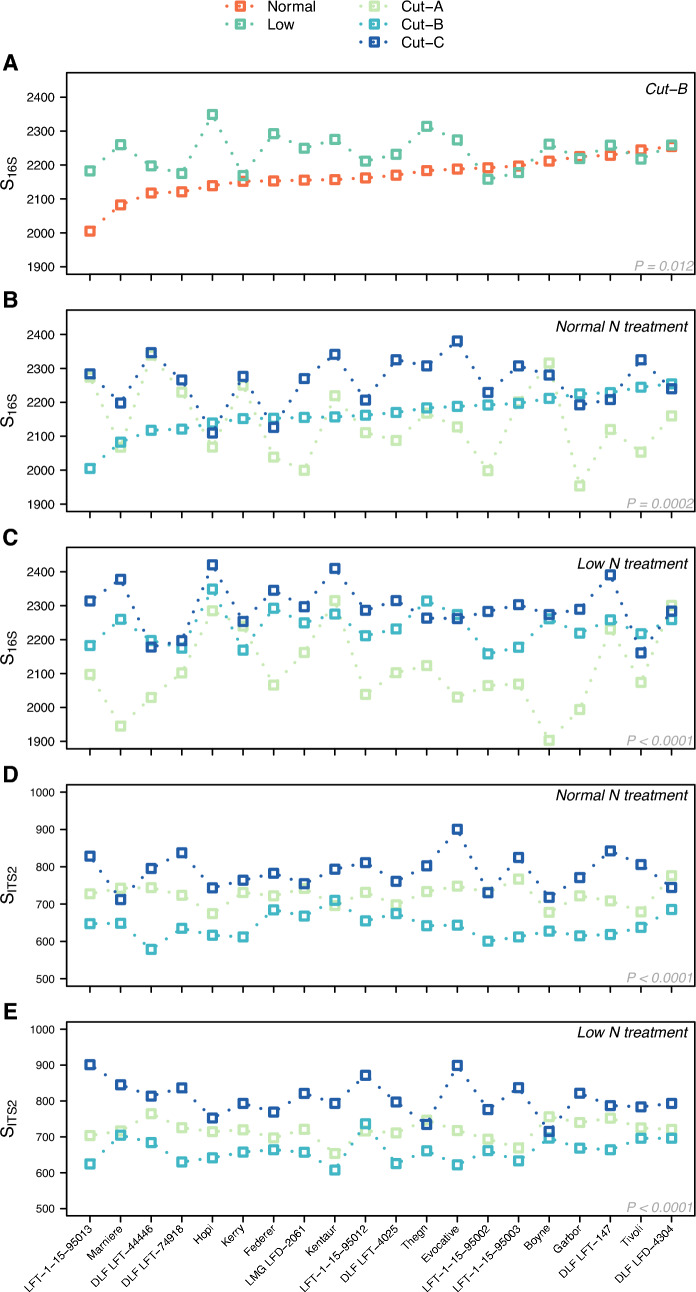
Species richness for bacteria and fungi. Comparison of bacterial species richness (S_16S_) for all ryegrass varieties as function of nitrogen treatment (**A**). The bacterial species richness (S_16S_) for normal (**B**) and low nitrogen treatment (**C**) across the three seasonal cuts. The fungi species richness (S_ITS2_) for normal (**D**) and low nitrogen treatment (**E**) across the three seasonal cuts. The *P*-values are from ANOVA testing the effect of treatment (**A**), and seasonal cuts for normal (**B**,**D**) and low nitrogen supplement (**C**,**E**).

The within-sample bacteria and fungi diversity varied across season (Supplementary Tables S7, S8) with the highest species richness latest in the season for most perennial ryegrass varieties (Fig. 3). For bacteria, only samples collected in July (cut-B) showed that species richness was influenced by different levels of nitrogen treatment, where the samples with lower nitrogen supplement had higher species richness (*P* = 0.012, Supplementary Table S6). Separation of soil samples by seasonal cut, replicate, horizontal position, treatment, and ryegrass variety using bacterial and fungal OTUs is presented in Supplementary Fig. S2 and S3, respectively. Within-sample complexity did not differ significantly between ryegrass varieties or their ploidy when tested within collection time points or nitrogen treatment level (Supplementary Table S6).

Across all samples, within the three seasonal collection time points, we observed vast interactions between the relative abundances among bacterial and fungal OTUs, where the evolution through the season was also evident (Supplementary Fig. S4). Correlation network analyses revealed positive and negative connections not only within the bacterial and within the fungal taxa, but also between bacteria and fungi (Fig. S4).

### Diversity within the soil microbiome

**Figure 4:**
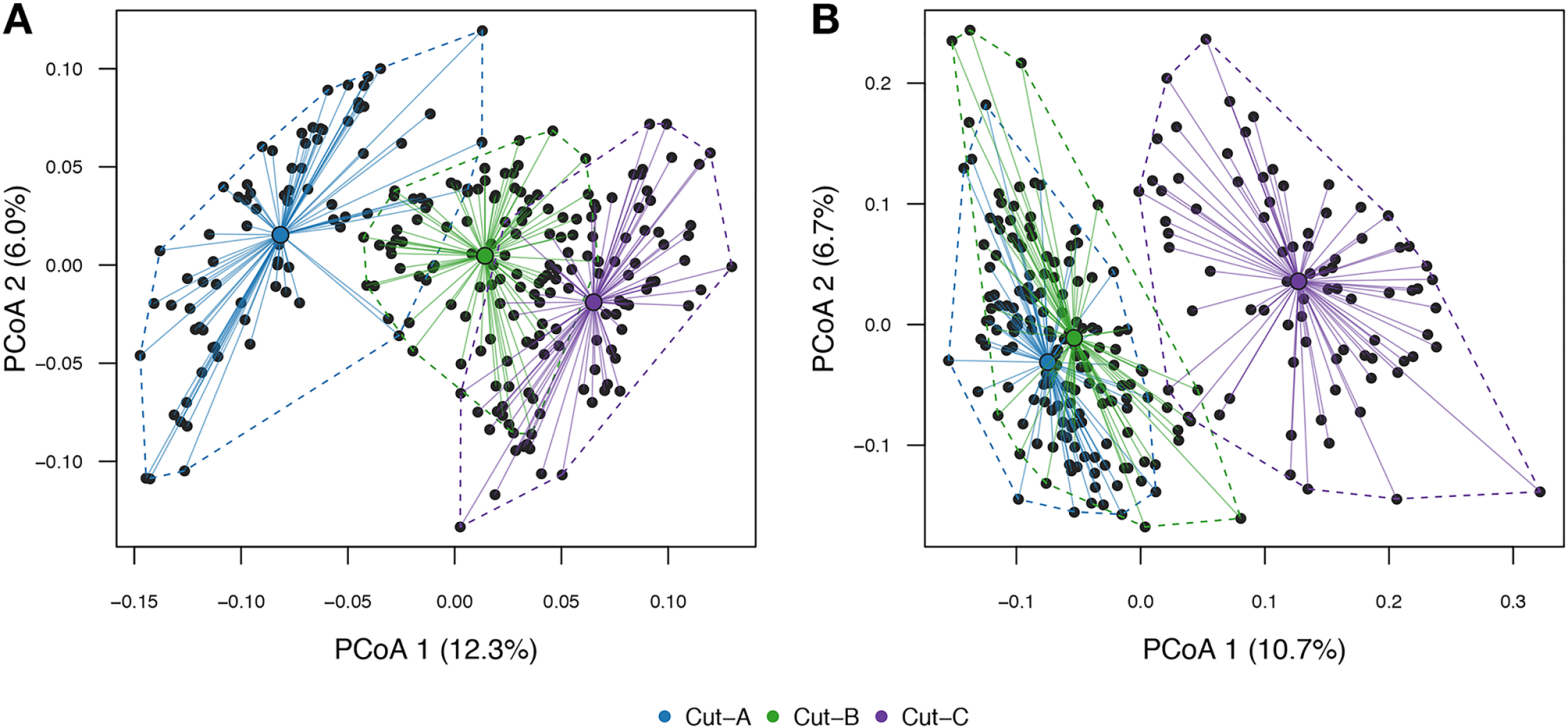
Principal coordinates 1 and 2 from PCoA of bacterial (**A**) and fungal (**B**) OTU communities. The color coding indicates which seasonal cut each sample belongs to. The centroid of each group is shown as larger dot in its respective color (see legend). The percent variation explained by the first two components is indicated on the axes.

We computed distance matrices adjusting for OTU abundance and phylogenetic distance among OTUs to estimate the across sample diversity. Principal coordinate analysis showed a clear separation of samples obtained from the different collection time points (Fig. 4). Next, we partitioned the variation in the soil microbiome diversity (Table 1) and found that 30.5% of the total variation in the bacterial microbiome could be explained by the experimental setup, with the largest fraction of variation explained by collection time point, i.e. seasonal cut (13.7%, *P* = 0.001), followed by ryegrass variety (8.15%, *P* = 0.001). Similarly, 27.70% of the variation in fungal microbiome diversity could be explained by the experimental variables, where 11.92% (*P* = 0.001) could be explained by the three collection time points and 7.87% by ryegrass variety (*P* = 0.001). Comparison of the distance matrices based on 16S OTUs and ITS2 OTUs for seasonal cut A, B and C are presented in Supplementary Fig. S5.

**Table 1:** Partitioning bacterial and fungal microbiome diversity into different sources of variation. The results are obtained from permutational multivariate ANOVA based on 999 permutations.

	Df	Sums of squares	F statistic	Variance explained (%)	*P* value
Bacteria					
Treatment	1	0.1411	4.7523	1.55	0.001
Replicate	1	0.3226	10.8696	3.54	0.001
Horizontal position	3	0.3319	3.7279	3.65	0.001
Seasonal cut	2	1.2456	20.9838	13.68	0.001
Ryegrass variety	19	0.7418	1.3154	8.15	0.001
Residuals	213	6.3220			
Fungi					
Treatment	1	0.3906	4.5729	1.56	0.001
Replicate	1	0.8670	10.1514	3.45	0.001
Horizontal position	3	0.7311	2.8534	2.91	0.001
Seasonal cut	2	2.9995	17.5602	11.92	0.001
Ryegrass variety	19	1.9812	1.22	7.87	0.001
Residuals	213	18.1916			

### Variation in soil microbiome and plant dry matter yield

Based on dry matter yield of a total of 64 ryegrass varieties, we estimated the proportion of dry matter variation attributable to the different ryegrass varieties (i.e. broad-sense heritability, ${\widehat{H}}^{2}$) to ${\widehat{H}}^{2}=0.37$, with strong evidence of variety-by-treatment-by-seasonal cut interaction (*P* = 1.87 × 10^−6^; Supplementary Table S9 for fixed effect analysis). For the subset of 20 ryegrass varieties used for the soil microbiome analysis, the heritability estimate was ${\widehat{H}}^{2}=0.37$.

**Figure 5:**
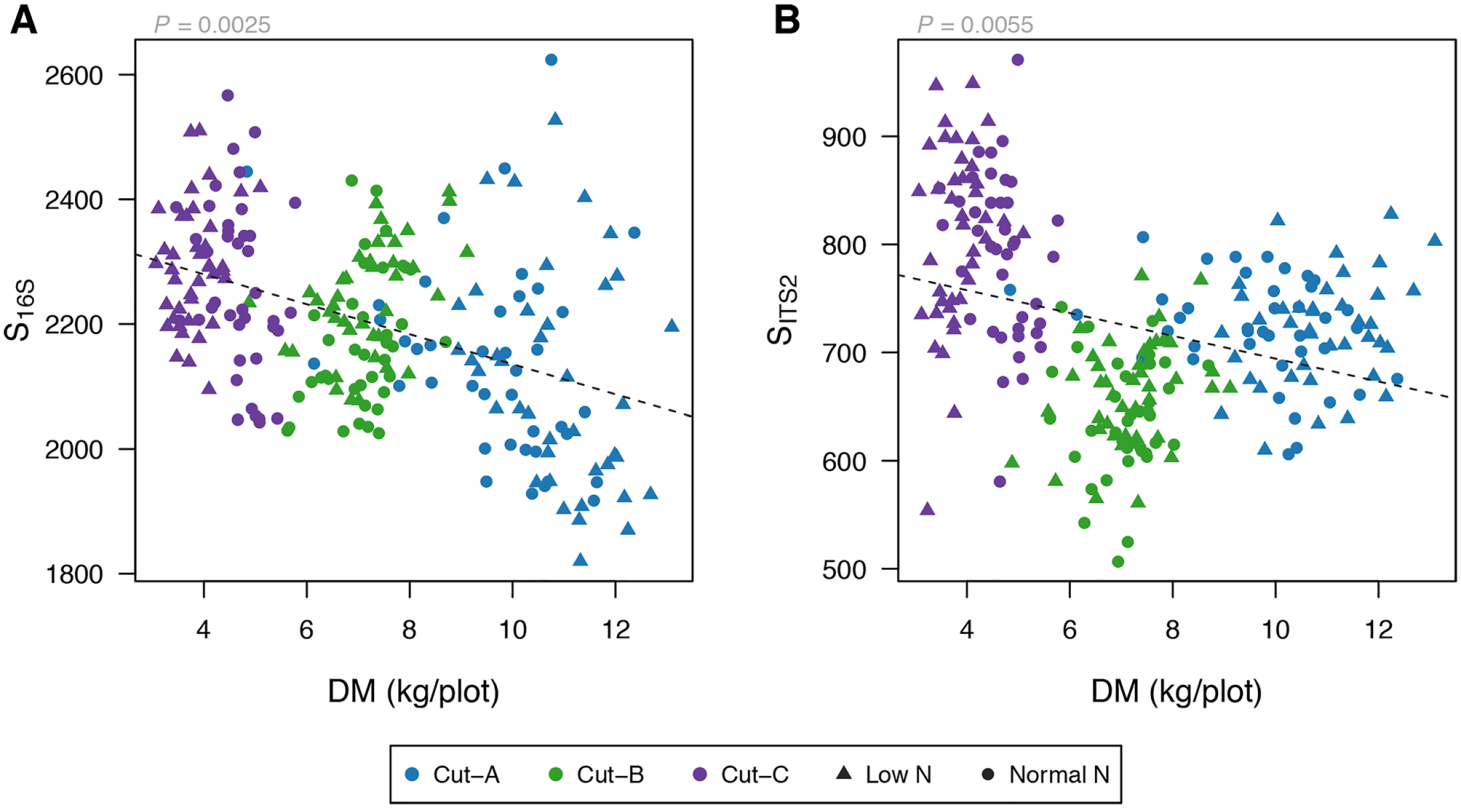
Bacteria (${S}_{16S}$) and fungi (${S}_{ITS2}$) species richness as a function of ryegrass dry matter (kg/plot). The *P* value for the regression coefficient is shown above each panel.

We found that bacteria and fungi species richness was negatively associated with ryegrass dry matter yield (Fig. 5). Similar effects were found for Shannon’s Index (not for fungi) and Faith’s phylogenetic diversity (Supplementary Fig. S6). Contrast plot of ryegrass dry matter (DM kg/plot, points) as function of replicate, horizontal groups, seasonal group and by low (n) or normal (N) nitrogen treatment are presented in Supplementary Fig. S7.

Because species richness was strongly associated with ryegrass dry matter, we then investigated if any OTUs were associated with ryegrass dry matter across season and nitrogen treatment. We identified four bacteria and three fungi OTUs that showed statistical association with ryegrass dry matter (Table 2, detailed in Supplementary Tables S10, S11). The bacteria OTUs belong to the phylum *Actinobacteria*, *Proteobacteria* and *Acidobacteria*, one fungi OTUs belongs the phylum *Basidiomycota*, whereas the other two had no BLAST hit. OTU richness was associated with decreased plant mass, except for bacteria from the order *Solirubrobacterales* (OTU_4991), which was associated with increased plant biomass (Supplementary Figs. S8, S9).

**Table 2:** Significant associations between ryegrass dry matter and bacteria and fungi OTUs. Full list is available in Supplementary Tables S10 and S11 for bacteria and fungi, respectively. FDR: false discovery rate.

OTU id	Domain	Phylum	Class	Order	Family	Genus	Species	FDR *P* value
OTU_1062	*Bacteria*	*Acidobacteria*	*Subgroup_6*					0.00634
OTU_1168	*Bacteria*	*Acidobacteria*	*Subgroup_6*					0.00713
OTU_4991	*Bacteria*	*Actinobacteria*	*Thermoleophilia*	*Solirubrobacterales*	*Elev-16S-1332*			0.0227
OTU_1496	*Bacteria*	*Proteobacteria*	*Gammaproteobacteria*	*Enterobacteriales*	*Enterobacteriaceae*	*Buchnera*		0.0228
OTU_580	*Fungi*	No BLAST hit						0.0009
OTU_933	*Fungi*	No BLAST hit						0.0467
OTU_99	*Fungi*	*Basidiomycota*	*Tremellomycetes*	*Tremellales*	*Incertae sedis Tremellales*	*Tetragoniomyces*	*Tetragoniomyces uliginosus*	0.04673

## Discussion

Despite the increasing number of soil microbial community studies, still limited information is available related to how the microbial composition variation contributes to herbage production across seasons and production years. In the present study, we characterized the soil microbial community of perennial ryegrass under field conditions in order to investigate to what extent the soil microbiome was influenced by plant genotype, whether the microbial community was stable across the season and if the microbiome diversity was altered by a 20% difference in applied nitrogen levels. We focused on the top 15 cm, the soil layer with the largest plant biomass^31,32^, with previous reports observing no significant effect of seasonality on perennial ryegrass root counts in soil layers deeper than 7 cm^33^. We identified several bacteria and fungi OTUs and showed that seasonality and ryegrass variety were the two most important factors in explaining the microbial diversity. We observed strong variety-by-environment interaction for ryegrass dry matter yield and this was associated with bacterial and fungal OTU richness. Finally, we identified seven OTUs associated with variation in ryegrass dry matter yield.

Based on a total of 240 soil samples, we identified over 6000 bacterial and close to 3000 fungal OTUs. Over 80% of the bacterial OTUs were found within four phyla, where *Actinobacteria* clearly dominated through the whole season, in all three collection time points. This is similar to what was observed in other studies^34,35^. The soil samples were likewise dominated by the fungal phyla *Ascomycota*, as expected based on previous reports^36^. Notable is however, that 30–40% of all fungal OTUs could not be annotated due to lack of BLAST hits. While this limits the interpretation of results, it also highlights the ample amount of research and work needed in this research field.

The major source of soil microbiome variation was attributable to the three sample collection time points (Table 1, Supplementary Tables S4, S5), which is most likely due to abiotic environmental changes caused by natural seasonality. Although both bacterial and fungal communities were influenced by the collection time points, their evolution through the season showed differences related to abundance, positive and negative associations, but also with respect to the evolution of the community across the season (Fig. 4, Supplementary Fig. S4). While bacteria showed a gradual change through the collection time points (Fig. 4A), the notable change in the fungal community was observed late in the season, at the third cut (Fig. 4B). Even with collection time point accounting for the largest amount of variation in the soil microbiome of perennial ryegrass, the individual varieties had a strong influence on this effect (Fig. 3).

In contrast to other studies where increased nitrogen supplements led to reduced bacterial richness^37–39^, we did not observe this trend (Supplementary Fig. S2, S5, and Supplementary Table S7). Although we did identify a significant effect of nitrogen treatment on species richness for cut-B, in July (Fig. 3, Supplementary Table S6), we noticed rather strong variety-specific effects of the nitrogen treatment (Fig. 3, Fig. S5), in accordance with a previous report for barley^39^. Wang et al. observed no significant impact of nitrogen treatment on perennial ryegrass soil microbiome structure and composition across different farm niches^40^, in an experiment comparing three nitrogen application rates of 0, 150 and 300 kg ha^−1^ year^−1^. The soil samples were collected from similar depth level, but the differences between nitrogen treatments are significantly larger compared to our study. The reduction in the amount of applied N was only around 20% in our study. Wang et al. noticed however a significant reduction of the microbiome network connectivity in soil. Variety specific effects could explain the differences observed between these studies, as our results indicate that plant genotype affects both the bacterial and fungal soil community. We observed that the soil microbiome of different varieties responded differently to the two nitrogen levels across the three cuts. For example, as seen in Fig. 3 for cut-B, the soil microbiome of the variety Kerry showed no difference between the two nitrogen levels, while a clear response to nitrogen treatment was observed for the variety Hopi. This suggests a different potential for the genetic background of different varieties, which could be further utilized in breeding. Escudero-Martinez et al. identified genomic loci having a major effect on the barley rhizosphere microbiome composition, and with no association to the investigated root phenotypic traits^41^. One of these loci is located on chromosome 3H, with three candidate genes suggested to control the heritable component of the barley rhizosphere microbiome. Furthermore, individual genes were observed to have different effects on fungi and bacteria. The recent advances in elucidating the perennial ryegrass genome^42^ will facilitate the identification of genomic regions involved in shaping its microbiome.

Different patterns were observed in case of both bacteria and fungi through the season depicted by the three cuts, depending on variety (Fig. 3, Supplementary Fig. S2). We were not able to directly identify within-variety differences in microbiome community composition (Supplementary Table S7) and this is most probably due to the limited per-variety samples with only two biological replicates. However, when comparing the entire soil microbiome community across the different ryegrass varieties, it was evident that the different varieties captured a significant proportion of the total microbiome variation (Table 1). Previous studies have also reported correlations between soil microbiome and plant genotype in other species^39,43–46^, while a cultivar effect was reported for the perennial ryegrass seed microbiome^47^. Future studies based on perennial ryegrass should be focused on elucidating the specific genetic elements involved in shaping its soil microbiome, to develop genetic markers to be employed in breeding.

Additional support for the existence of host genotype–microbiome interactions was obtained by incorporating plant phenotypic measurements of plant biomass. We estimated the proportion of phenotypic variation that could be explained by the different ryegrass varieties to 0.37 (independent on whether we used the entire dataset of 64 varieties or the subset of 20 varieties) and found a negative correlation between bacterial and fungal richness and ryegrass dry matter yield (Fig. 5). These results suggest that the local soil microbiome was associated with plant genotype and with plant phenotype; here, plant biomass.

Seven OTUs were statistically associated with ryegrass dry matter yield (Table 2), where six of them showed a negative association. Two of these OTUs belong to the phylum *Acidobacteria,* one of the most dominant soil bacteria detected in a wide range of different soil habitats^48–52^. In particular subgroup 6 has been shown to respond to high content of soil Ca, Mg, Mn, and B^53^. Our results suggest their presence and/or activity in the soil to be less beneficial for the plants. Another OTU belongs to the *Enterobacteriaceae* family, which is a class of bacteria known for its many pathogens, but also for harmless symbionts. The identified OTU belongs to the *Buchnera* genus, which is not well described. But given the observed negative association, it is less likely that these bacteria are neutral to the plant. The fungal OTU_99 belongs to *Tetragoniomyces uliginosus* and has previously been described to have a mycoparasitic lifestyle^54^, which probably explains the negative correlation observed in this study. Although the two other fungi OTUs with significant negative correlation to dry matter yield are unknown at this point, our results suggest a negative effect of these on plant performance. The only OTU found to be positively associated with increased ryegrass dry matter (Supplementary Fig. S8) belongs to the *Actinobacteria* order *Solirubrobacterales*. This group of bacteria has previously been described as cosmopolitan and widely present in the soil^55^, more abundant in the soil during the dry season^56^ and more abundant in conventionally managed soil compared to organic agroecosystem^57^. Also, it was previously associated with transportation of chemical contamination^58^ and with the ability of decomposing lignin^59^. Some endophytic members of this group were also described^60^. *Solirubrobacteraceae* family showed different correlation patterns to specific metabolites in Arabidopsis accessions differing in resistance to *Fusarium oxysporum*^15^ and was found to supress common scab disease of potato^61^. Although limited information is available related to the *Solirubrobacterales* members, a recent review summarizes the direct and indirect plant growth promoting role of *Actinobacteria*, highlighting the potential of some of the better characterized strains as natural fertilizer and pesticides^62^. Our results suggest that the presence of OTU_4991 in the soil microbiome has a positive effect on perennial ryegrass yield.

The limitations of the present study need to be considered when interpreting the presented results. First, the study was performed at a single location in Denmark, while a replicate in another location with a similar environmental profile would be advantageous. Second, the experimental test field had a vertical gradient which could have influenced the soil humidity and thereby the plants’ access to water. We have accounted for the exact location in the field in the statistical analysis, however, micro-environmental variability in water access could still play a role in both plant dry matter and microbial composition. Third, sequencing was performed for two biological replicates, which limits the statistical power and the accuracy with which we can estimate parameters. Despite the practical limitations posed especially by the only two biological replicates for each nitrogen treatment, our results are promising and lay foundation for future large studies to uncover the specific components of the microbiome positively affecting plant traits of interest. Biostimulants based on nitrifying bacteria have already been shown to exhibit positive effects on perennial ryegrass performance, even under nutritional stress conditions^63^. Large scale studies, with sufficient biological replication and comprising a large number of varieties ideally to be tested in multiple locations should be designed to confirm the effects of specific soil microbiome components on plant performance.

There was a clear difference in the development and evolution of fungal and bacterial community through the season, with positive and negative interactions between components of perennial ryegrass soil microbiome, within and across phyla. Our results indicate that the plant variety has a strong influence on the soil microbiome, while the microbiome has a strong influence on plant variety performance. This opens for possibilities towards developing new varieties harnessing the potential and attributes of the microbiome. Moreover, the potential for continuation of the perennial ryegrass bacterial microbiome from seed, through plant maturation and to seed was suggested, highlighting its hereditary potential^64^. It is envisioned that a specially tailored microbiome could be used as an enhancer for traits of interest, repressor or natural pest control, natural fertilizer, promoting a more sustainable and efficient agriculture and opening for the next Green Revolution. There is a clear need for further studies in these directions. The cooperative modulating role of the host and environmental variables emphasizes the importance of these variables for future development and application of synthetic microbiomes.

## Material and methods

### Field trial

This study was based on a larger field experiment which included a total of 64 ryegrass varieties. The soil samples were collected from plots established at Tystofte Foundation, located in the south-western region of Zealand, Denmark. The soil in this region is characterized as fine sandy clay^65–69^, with the composition of 10–15% clay, 0–30% silt, 40–90% fine sand, 55–90% sand and less than 10% humus. The seeds from 64 perennial ryegrass varieties were sown in August 2016, alternating a normal nitrogen treatment and an approximately 20% lower nitrogen treatment, using inorganic N fertilizer (Table 3), with two replicates for each treatment. Each variety was sown in a plot of size 1.5 m × 10 m. In the following year, after the plants were established, a total of twenty (five diploid and fifteen tetraploid) varieties were subjected to the soil microbiome study. The field layout and location of the sampled varieties within the field are illustrated in Supplementary Table S1.

**Table 3:** Amount of nitrogen fertilizer applied to the field trial.

Amount of N applied (kg/ha)	Low N (n)	Normal N (N)
At the start of plant growth	112	152
After 1st cut	84	114
After 2nd cut	56	76
After 3rd cut	28	38
Total	280	380

### Soil sample collection

This study considered 20 of the 64 ryegrass varieties (Supplementary Table S1). Three collection time points were selected as the day after the first, second, and third cut of the plants (cut-A: 24.05.2017, cut-B: 07.07.2017, cut-C: 17.08.2017). This study included thus 20 varieties × 2 treatments × 2 biological replicates × 3 sample collection time points; in total 240 soil samples. The soil samples were collected using a sampling tube, to a depth of 15 cm, from five points for each plot: the middle and 40 cm distance on the diagonals from each corner; these were afterwards mixed to represent the plot, as a sample for one variety. The soil samples were stored at − 20 °C, then freeze-dried. Roots were removed through agitation and sifting.

### Perennial ryegrass dry matter yield

Dry matter content was measured using near infrared reflectance spectroscopy, and DM kg/plot was computed from dry matter content and total green mass yield harvested, as previously described^70^. The phenotyping data was kindly provided by the breeding company DLF, who performed the phenotyping according to their custom protocol as part of the GreenSelect project, funded by the Green Development and Demonstration Research Programme.

### DNA extraction and sequencing

Total DNA was extracted from 0.25 g of soil using the DNeasy PowerLyzer PowerSoil kit (Qiagen) according to the manufacturer’s instructions. DNA concentration was assessed with Quant-iT PicoGreen dsDNA assay kit (Invitrogen, Oregon, USA). The sequencing was performed as custom paid service by Novogene (Novogene Corporation) using their TruSeq^®^ DNA PCR-Free Library Prep workflow and the Illumina HiSeq platform, resulting in 250 bp paired-end reads. Region V3-V4 with a fragment length of 466 bp was targeted for the bacterial 16S amplicon sequencing and the ITS2 region with a fragment length of 350 bp was used to target fungi.

### Sequence analysis and OTU identification

Paired-end reads were merged using FLASH (V1.2.7)^71^ and chimera sequences were detected with the UCHIME algorithm^72,73^. 30.2% of the 16S and 18% of the ITS2 sequences were chimera or low quality (Phred score < 20) and were removed (Supplementary Tables S2, S3). Sequences with 97% nucleotide similarity were clustered into operational taxonomic units (OTUs) using Uparse software^74^. For the 16S data, sequences were screened for annotation against the SSUrRNA database of the SILVA Database^75^ using Mothur^76^ with a threshold of 0.8–1 for taxonomic ranks. For the ITS2, sequence annotation was obtained by BLAST with Qiime^77^ and the Unit database^78^.

The raw OTU tables were scaled to the minimum number of reads across all samples (16S = 29,710 and ITS2 = 37,959) prior to statistical analyses, using the “rarefy” approach. OTUs with presence less than 0.1% sequences across all *N* samples were filtered out resulting in 6092 bacterial OTUs (based on 16S rRNA) and 2907 fungi OTUs (based on ITS2). The relative abundance within sample was computed for each OTU. To get the phylogenetic relationship among the OTUs (16S and ITS2 separate) we used MUSCLE^79^ for multiple sequence alignment and to construct a UPGMA (Unweighted Pair Group Method with Arithmetic Mean) phylogenetic tree.

### Within sample complexity

Different measures of within sample OTU diversity (α-diversity) were computed from the bacteria and fungi OTU tables using the R packages RAM^80^ and picante^81^. We computed the OTU richness (*S*), Shannon’s Index (*H*), and Faith’s phylogenetic diversity (*PD*).

We compared the within sample diversity metrics (${y}_{\alpha }$) across ryegrass varieties (${y}_{\alpha }=rep+horz+variety+e$), ryegrass ploidy level (${y}_{\alpha }=rep+horz+ploidy+e$), nitrogen treatment level (${y}_{\alpha }=rep+horz+variety+treatment+e$) and seasonal cuts (${y}_{\alpha }=rep+horz+variety+cut+e$) using linear models, and comparing the full model to a reduced model neglecting the term of interest. For all comparisons we included a replicate effect (*rep*) and the horizontal position effect (*horz*) corresponding to the position in the field.

We conducted a combined correlation network analysis by computing Spearman’s correlation of the relative abundance among all bacteria and fungi OTUs. We only considered correlations that were significant (Bonferroni adjusted *P* value < 0.001) and had correlation coefficients below − 0.5 or above 0.5. The correlation networks were visualized with iGraph^82^.

### Across sample complexity

While the α-diversity measures the within-sample diversity, the β-diversity is the OTU diversity across samples. First, we computed a distance metric over the OTUs using the generalized UniFrac distance^83^ using the R package GUniFrac^84^, which is a distance measure that incorporates the genetic distance of the OTUs in each sample to the OTUs in all the other samples. We then performed a permutational multivariate analysis of variance—PERMANOVA, implemented in the R vegan package^85^ to partition the variation within the distance matrix to seasonal cuts, replicate, in field location/horizontal placement, treatment and ryegrass variety. The PERMANOVA tests for differences in the centroid and variances among the groups (thus, seasonal cuts, replicate, horizontal placement, treatment and ryegrass variety) and also provides estimate of proportion of total variance explained by each factor. The results were visualized by plotting the first two principal coordinate axes from the principal coordinate analyses (PCoA).

### Association between microbiome diversity and ryegrass dry matter

We estimated the proportion of total phenotypic variation (of dry matter yield) explained by ryegrass varieties (${\widehat{H}}^{2}$) by fitting a linear mixed model with the R package lme4^86^ assuming independence among the ryegrass varieties. We obtained estimates of ${\widehat{H}}^{2}$ using the entire data set of 64 ryegrass varieties and for the subset of 20 varieties used in the soil microbiome study by fitting $y=hrz+rep+cut+tr+cut:tr+id+id:tr:cut$, where $y$ was a vector of dry matter content, the horizontal groups ($hrz$), replicate ($rep$), seasonal cut ($cut$), treatment ($tr$), and the interaction between cut and treatment ($cut:tr$) were considered as fixed effects. The ryegrass varieties ($id$) and the interaction between ryegrass varieties and treatment and seasonal cut ($id:tr:cut$) were considered as random effects. The broad sense heritability was estimated as ${\widehat{H}}^{2}=\frac{{\widehat{\sigma }}_{id}^{2}+{\widehat{\sigma }}_{id:tr:cut}^{2}}{{\widehat{\sigma }}_{id}^{2}+{\widehat{\sigma }}_{id:tr:cut}^{2}+{\widehat{\sigma }}_{e}^{2}}$, where ${\widehat{\sigma }}_{id}^{2}$ is the variance among ryegrass varieties, ${\widehat{\sigma }}_{id:tr:cut}^{2}$ is the variety-treatment-cut interaction variance, and ${\widehat{\sigma }}_{e}^{2}$ is the residual variance.

Finally, we investigated if any bacteria or fungi OTUs were associated with variation in ryegrass dry matter. We fitted one OTU at a time (${otu}_{i}$) specified as a fixed effect $y={otu}_{i}+hrz+rep+cut+tr+cut:tr+id+id:tr:cut$, and compared the model to one neglecting the OTU using a likelihood ratio test. Following, all *P* values were adjusted for multiple testing using a false discovery rate (FDR) < 0.05 as significance threshold.

### Supplementary Information

Supplementary Tables.

Supplementary Figures.

## Data Availability

Sequencing data is deposited in the EMBL-EBI/MGnify and will be made publicly available upon publication of the study.

## Abbreviations

OTU: Operational taxonomic unit
G × E: Genotype by environment interactions
