# Supplementary material for: The soil microbiome of Lolium perenne L. depends on host genotype, is modified by nitrogen level and varies across season
Source: Sci Rep. 2024 Mar 8;14:5767. doi: 10.1038/s41598-024-56353-2 (PMC10923896; doi:10.1038/s41598-024-56353-2)
Supplement: Supplementary file 2 — Supplementary Figures. [file 41598_2024_56353_MOESM2_ESM.docx]

**Figure S1.** Comparison of α-diversity metrics. The figure compares OTU richness (S), Shannon’s Index (H) and Faith’s Phylogenetic Diversity metric (PD) for bacteria (16S) and fungi (ITS2) OTUs.


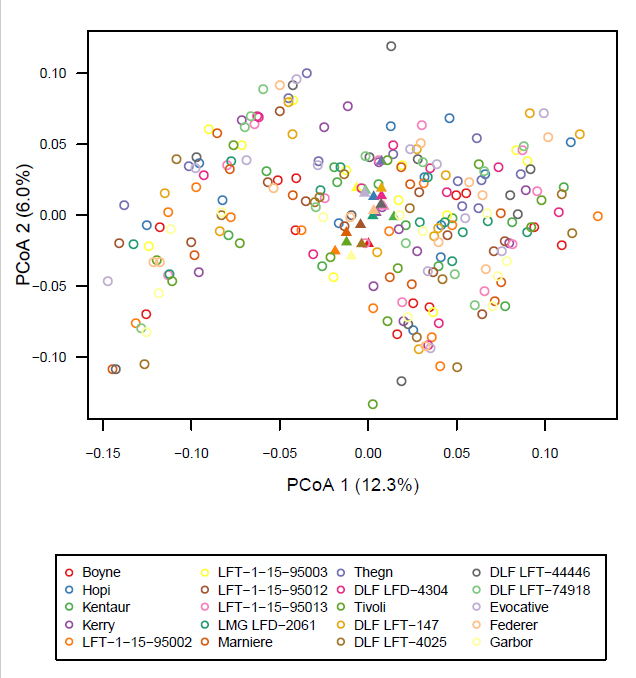


**Figure S2.** Separation of soil samples by seasonal cut, replicate, horizontal position, nitrogen treatment and ryegrass variety using bacterial OTUs. Ryegrass varieties are shown in different colors. The ryegrass variety accounts for 8.15% of the total variation (Supplementary Table S6). Triangles indicate within ryegrass variety centroid.


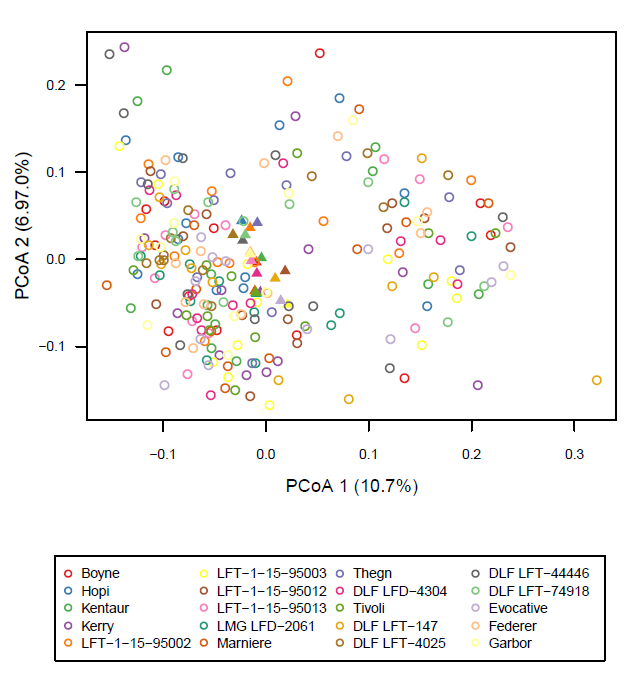


**Figure S3.** Separation of soil samples by seasonal cut, replicate, horizontal position, treatment and ryegrass variety using fungi OTUs. Ryegrass varieties are shown in different colors. The ryegrass variety accounts for 7.87% of the total variation (Supplementary Table S7). Triangles indicate within ryegrass variety centroid.


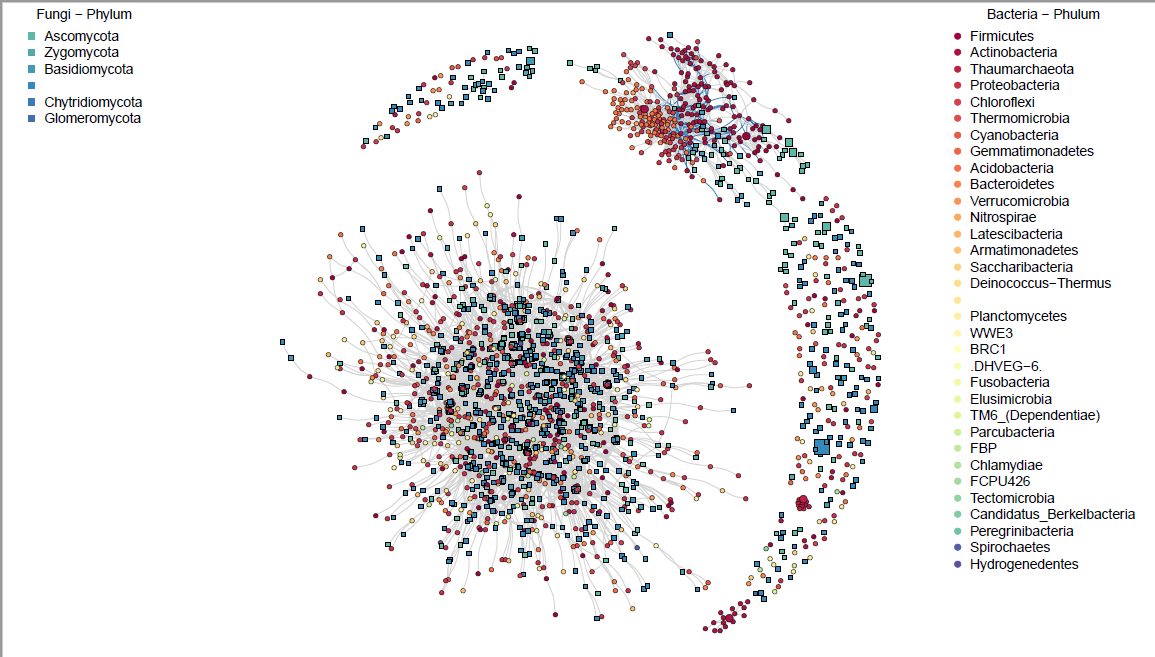


(a)


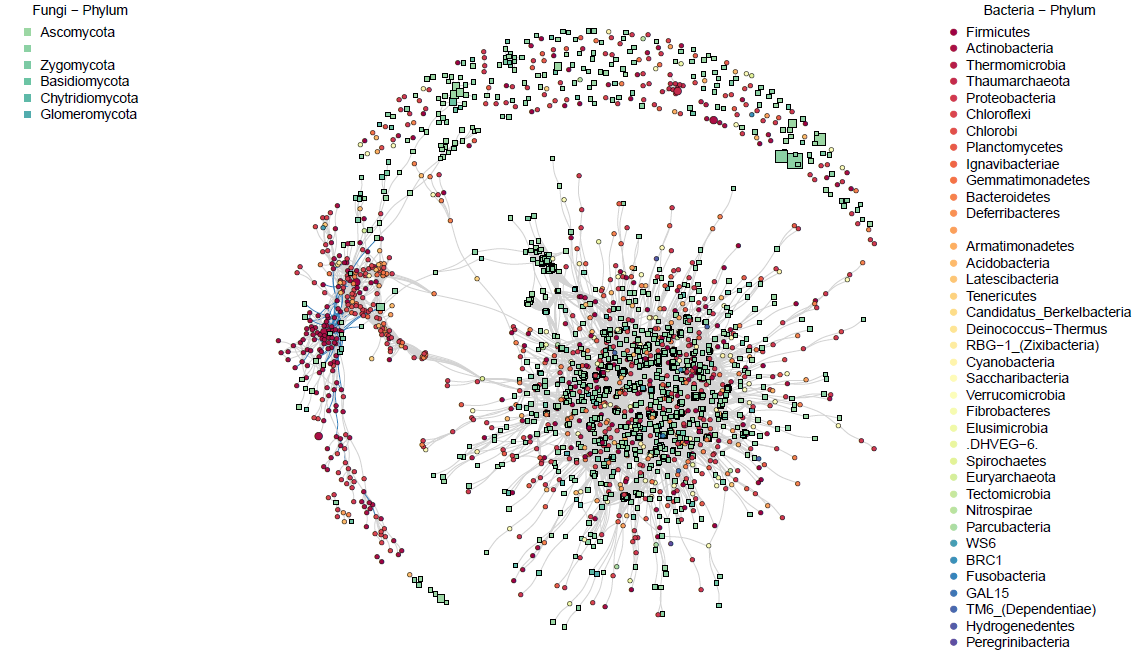


(b)


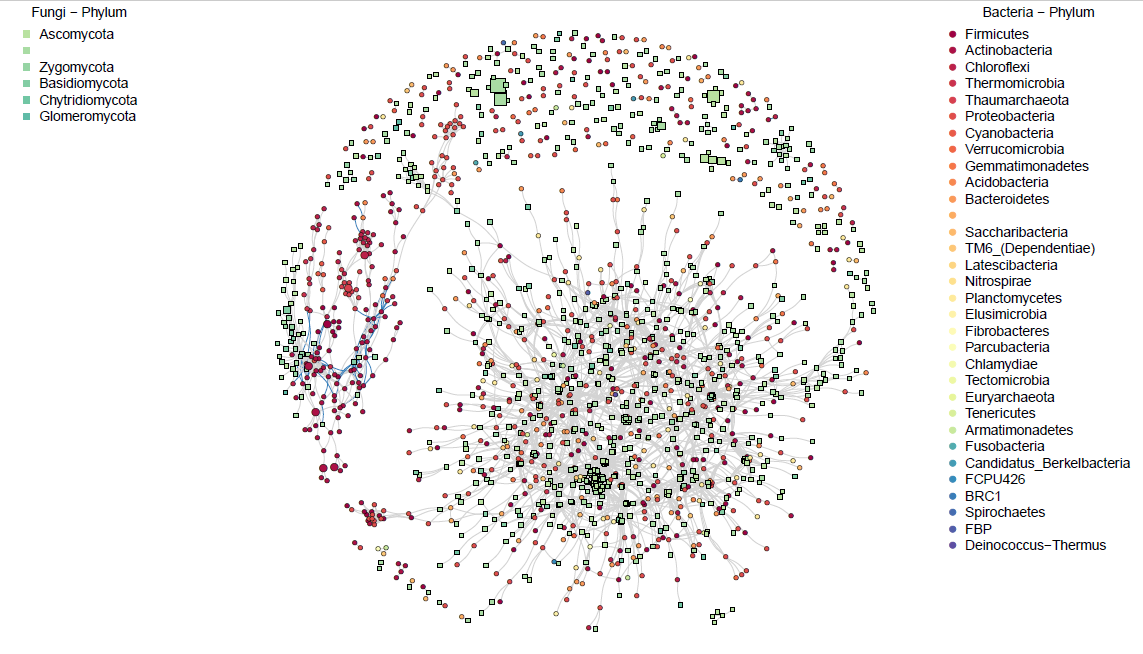


(c)

**Figure S4.** Correlation network analyses of the perennial ryegrass rhizosphere microbiome for the first cut A (a), second cut B (b), and third cut C (c), highlighting correlations above 0.7 and below -0.7, with an adjusted *p*-value of 0.001. Bacterial taxa are represented with circles, fungi with squares, with size of circles/squares corresponding to abundance. Grey lines represent positive connections, blue lines negative connections. Color codes are described in the legend. Color code with no description corresponds to unknown taxa.

**Supplementary Figure S5.** Comparison of the distance matrices based on 16S OTUs and ITS2 OTUs for seasonal cut A, B and C.

**
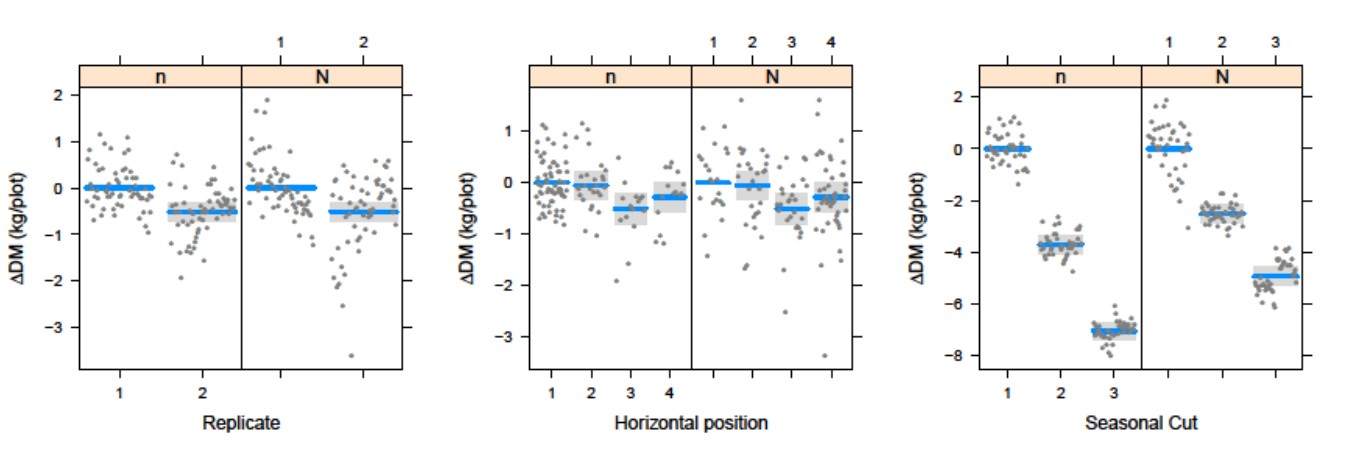
**

**Supplementary Figure S6.** Contrast plot of ryegrass dry matter (DM kg/plot, points) as function of replicate, horizontal groups and seasonal groups by low (n) or normal (N) nitrogen treatment (the reference value is by the first horizontal group). Blue bars represent the estimated coefficients from the linear mixed model, grey bands represent the confidence intervals.

**Supplementary Figure S7.** Association between ryegrass dry matter (DM, kg/plot) and within sample microbiome diversity measures (Species richness, S; Shannon’s Index (H); Faith’s Phylogenetic Diversity (PD)).

**
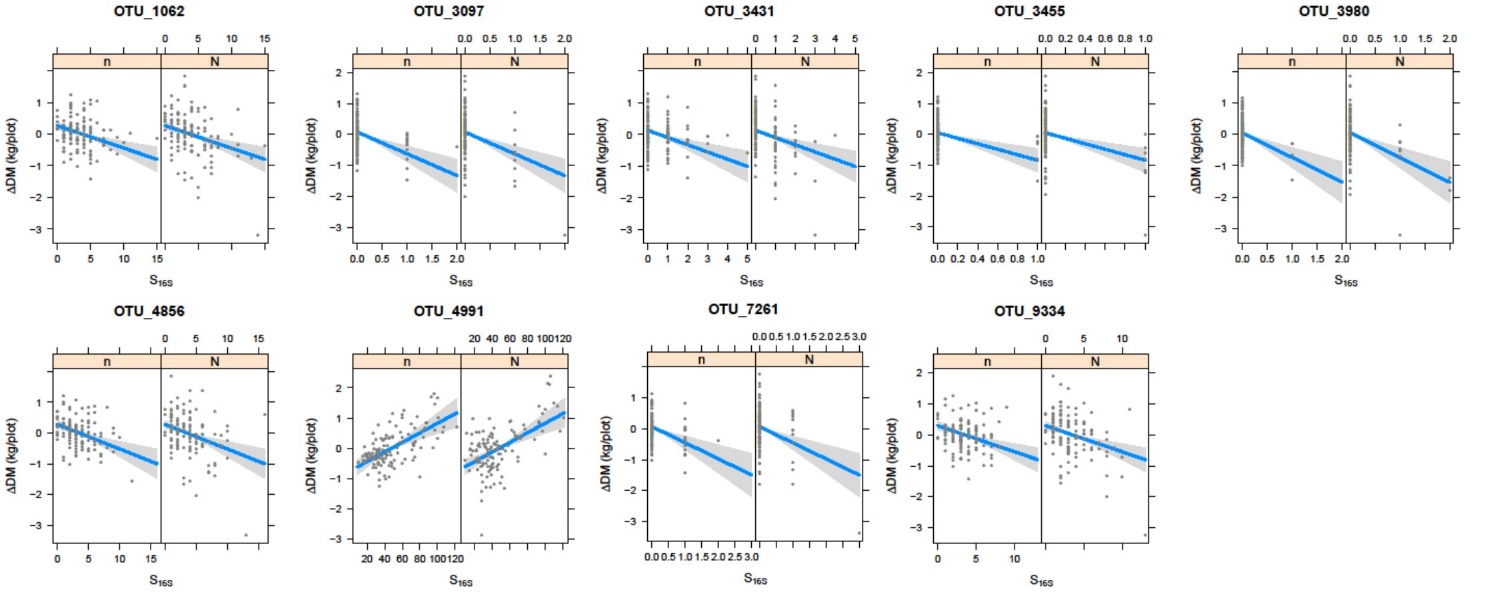
**

Figure S8. Contrast plot of ryegrass dry matter (DM kg/plot, points) as function of significant bacteria OTUs group by low (n) or normal (N) nitrogen treatment (the reference value is by the first horizontal group). Blue lines represent the estimated coefficients from the linear mixed model, grey bands represent the confidence interval.

**
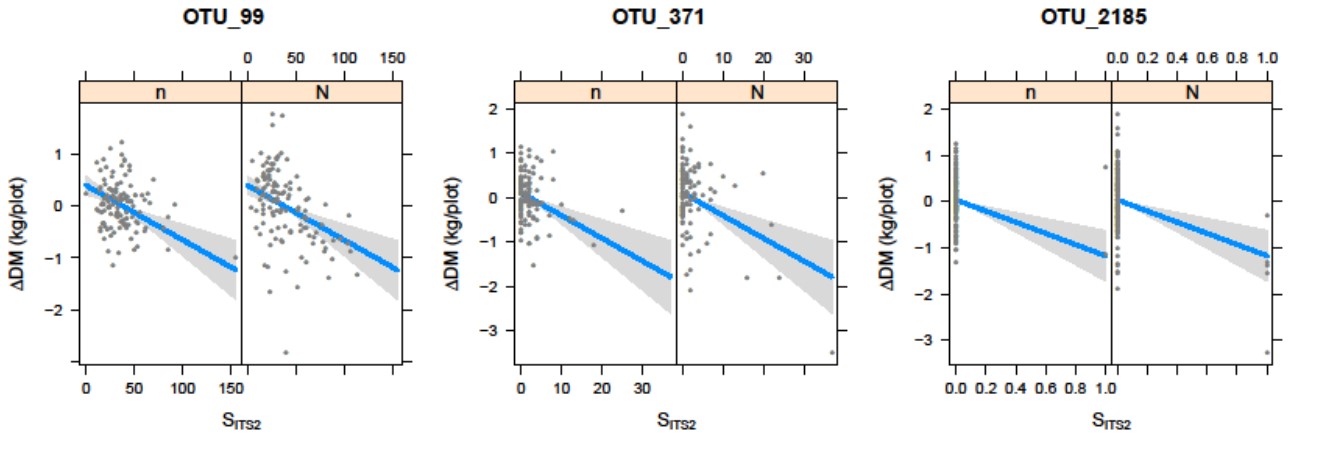
**

**Figure S9.** Contrast plot of ryegrass dry matter (DM kg/plot, points) as function of significant fungi OTUs group by low (n) or normal (N) nitrogen treatment (the reference value is by the first horizontal group). Blue lines represent the estimated coefficients from the linear mixed model, grey bands represent the confidence interval.
